# Prospective clinical validation of a novel artificial intelligence system for real-time detection of solid pancreatic masses during endoscopic ultrasonography

**DOI:** 10.1055/a-2701-6530

**Published:** 2025-10-13

**Authors:** Ji Young Bang, Adrian Săftoiu, Anca Udriștoiu, Lucian Gruionu, Elena Codruţa Gheorghe, Gabriel Gruionu, Jayapal Ramesh, Charles Melbern Wilcox, Shyam Varadarajulu

**Affiliations:** 1 6246 Digestive Health Institute, Orlando Health, Orlando, United States; 2 Medical Softverse SRL, Craiova, Romania; 3 Elias Emergency University Hospital, Carol Davila University of Medicine and Pharmacy, Bucharest, Romania; 4 91861 Faculty of Automation, Computers and Electronics, University of Craiova, Craiova, Romania; 5 91861 Faculty of Mechanics, University of Craiova, Craiova, Romania; 6 121532 Faculty of Medicine, University of Medicine and Pharmacy of Craiova, Craiova, Romania; 7 1772 Krannert Cardiovascular Institute, Indiana University, Bloomington, United States

## Abstract

**Background:**

Endoscopic ultrasonography (EUS) is the most sensitive modality for accurately establishing a tissue diagnosis in patients with solid pancreatic masses. However, small lesions can be challenging to detect, particularly for less experienced endosonographers. Therefore, outcomes of EUS are operator dependent. We validated the performance of novel artificial intelligence (AI)-enhanced EUS for detection of solid pancreatic lesions.

**Methods:**

In this single-center, prospective, nonrandomized, comparative study, high-risk patients aged ≥18 years referred for pancreatic cancer screening or with suspected (solid and cystic) pancreatic lesions owing to symptoms, radiological, or laboratory findings were evaluated in real time using AI-EUS software. The model included 32 713 EUS frames (training/testing phases) of normal, solid, and >10-mm cystic pancreatic lesions from 202 patients. Clinical validation was conducted prospectively when EUS findings were evaluated concurrently in real time by two independent expert examiners, one using conventional EUS and another with AI-EUS, both blinded to the alternative assessments. The primary outcome was detection of solid pancreatic masses.

**Results:**

308 patients were evaluated (January–July 2024). AI-EUS performance was not significantly different to that of conventional EUS performed by experts (97.1% vs. 100%; risk difference 2.9%, 95%CI –1.2 to 6.8; P = 0.25). Final pathology of 105 pancreatic solid masses revealed neoplasia in 93 (88.6%) and benign lesions in 12 (11.4%).

**Conclusion:**

The performance of AI-EUS was not significantly different to that of experienced endosonographers for detection and segmentation of solid pancreatic masses. By standardizing performance, AI-EUS may have the potential to optimize clinical outcomes in pancreatic cancer.

## Graphical Abstract

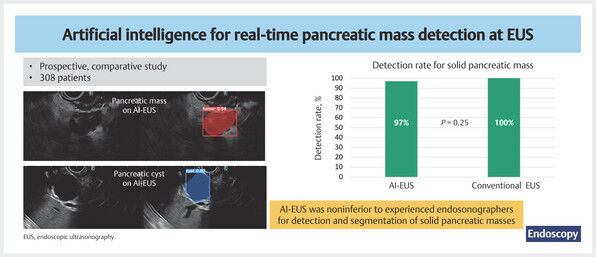

## Introduction

Pancreatic ductal adenocarcinoma (PDAC) is a highly lethal disease with an overall 5-year survival of approximately 11% after diagnosis 1 2 . This is because the majority of PDACs are diagnosed at an advanced, often locally unresectable or metastatic stage, which directly impacts survival. However, patients with resectable stage 1A PDAC have a 5-year survival of approximately 84% 3 , which underscores the potential for improved outcomes through early detection.

Unlike common cancers such as breast or colon, detection of PDAC can be difficult, even when symptomatic 4 . Current imaging modalities such as abdominal ultrasonography, computed tomography (CT), and magnetic resonance imaging (MRI) do not reliably detect small pancreatic cancers 5 6 7 . More recently, endoscopic ultrasonography (EUS) has emerged as a critical modality for imaging, staging, and diagnosis of pancreatic cancer. In a prospective study from the Netherlands of 139 high-risk patients who were screened by EUS and/or MRI, it was observed that while MRI was more sensitive for identifying cystic lesions, only EUS detected small solid pancreatic masses that were stage 1 PDAC or pancreatic intraepithelial neoplasia 7 . EUS can also visualize subtle parenchymal and ductal abnormalities more traditionally associated with chronic pancreatitis in up to a quarter of patients at high risk for PDAC 8 . When compared with CT, EUS has been shown to be superior for detection of small pancreatic neuroendocrine neoplasia, particularly lesions <10 mm in size 9 . Prospective studies have shown that outcomes for EUS-guided fine-needle biopsy of pancreatic masses are excellent, with diagnostic adequacy and accuracy of more than 95% and 90%, respectively 10 11 . Given these promising outcomes, the National Comprehensive Cancer Network recommends EUS guidance to be the preferred method for tissue acquisition in patients with suspected pancreatic cancer 12 . However, clinical outcomes of EUS and EUS-guided tissue acquisition are dependent on operator experience 13 14 15 . Consequently, there is a need for a more reliable method to standardize detection of solid mass lesions so that outcomes for PDAC can be improved.

Deep learning has been applied successfully to many clinical areas in digestive health 16 . In EUS, it has been utilized to monitor the quality of examinations 17 . Application of artificial intelligence-enhanced techniques for detection of pancreatic masses, particularly small lesions, could potentially improve clinical outcomes. Previously, we developed a deep learning algorithm based on convolutional neural networks (CNNs) for detection of solid pancreatic mass lesions, which, ex vivo, demonstrated an overall accuracy of 98.3% and area under the curve index of 0.98 18 . More recently, we further refined and updated pancreatic AI-enhanced EUS imaging analysis software (PANCRAIEUS; Medical Softverse SRL, Craiova, Romania) for real-time detection of both solid and cystic pancreatic lesions 19 . The objective of our present study was to validate the in vivo diagnostic performance of PANCRAIEUS for detection and segmentation of solid pancreatic mass lesions in real-time clinical practice.

## Methods

### Study design and participants

This was a prospective, single-center, nonrandomized, comparative study conducted in real time at a single tertiary referral center (Orlando Health, Orlando, Florida, USA). This study is part of an ongoing larger study (NCT06564571), which primarily focuses on computer-aided detection of pancreatic lesions according to guidance from the United States Food and Drug Administration (FDA). Therefore, differences exist between the current study design and study registration.

Inclusion criteria were consecutive inpatients and outpatients aged 18 years or older, scheduled for EUS evaluation of suspected pancreatic lesions owing to clinical symptoms, radiological, and/or laboratory findings, or patients at high risk for pancreatic cancer based on genetic and familial risk factors. Exclusion criteria were inability to undergo anesthesia, history of gastric surgery, pregnancy, inability to complete EUS examination owing to clinical instability or stricture of the esophagus, stomach, or duodenum. All authors had access to the study data, and have reviewed and approved the final manuscript.

### AI system

For development of PANCRAIEUS, we used two CNN architectures: Mask Region-based CNN (Mask R-CNN; Facebook AI Research [FAIR], Menlo Park, California, USA) and You Only Look Once, version 8 (YOLO v8.0; Ultralytics, London, UK). Categories of patients and video frames included are shown in Table 1 . The AI system was explicitly trained to recognize and categorize adjacent nonpancreatic structures (e.g. gallbladder, liver, kidney, spleen, vessels) as “none,” avoiding their misclassification as pancreas, cyst, or solid mass lesions. Thus, approximately 10% of training frames included adjacent structures classified explicitly as “none.” Early chronic pancreatitis and fatty pancreas were included broadly under the “pancreas” category.

**Table:** **Table 1** The distribution of patients and video frames between model training, performance testing, and clinical validation.

PANCRAIEUS	Number of patients				Number of frames			
	Total	Pancreas	Tumor	Cyst	Total	Pancreas	Tumor	Cyst
Model training	150	50	50	50	29 586	27 628	5512	3940
Performance testing	52	17	12	21	3127	2355	240	753
Clinical validation	308	99	105	104	10 064	6856	1762	1446
Final total	510				42 777			

Annotation and training for the PANCRAIEUS AI system involved a multistep approach to ensure high accuracy in segmenting and identifying pancreatic structures in EUS images. Further details are provided in the online-only **Supplementary Methods** and **Figs. 1.1s–1.3s** .

For subsequent model training ( **Supplementary Methods** ), annotated images were initially used to train the PANCRAIEUS system with detailed CNN architecture (Mask R-CNN) designed for accurate segmentation. Active learning was subsequently applied to expand the training set: the AI model, already trained on initial images, was used to segment additional images (150–250 per patient), which were then reviewed by the same EUS experts to rectify any errors. This iterative review process allowed for fast, refined, and accurate segmentation. In the final phase, corrected images were added to a dataset for retraining the model using streamlined YOLO v8-based CNN architecture, enabling faster processing across a comprehensive dataset of 150 patients included prospectively and consecutively (29 586 frames). This approach aimed to improve segmentation precision while maintaining computational efficiency, ensuring that PANCRAIEUS could effectively support accurate identification and analysis of pancreatic structures in the clinical settings.

For performance testing of PANCRAIEUS, we evaluated 52 different patients who were included prospectively and consecutively, yielding a total of 3127 frames. The patient cohort included 21 individuals with normal pancreas, 12 with cysts >10 mm, and 19 with solid masses. Further details of performance testing are provided in the **Supplementary Methods** , **Figs. 2.1s–2.6s** , and **Table 1s** . A four-class confusion matrix was individually calculated for each patient, confirming very low numbers of individual false-positive frames (cystic lesions or solid tumors) due to interference of adjacent structures. Representative adjudication examples are provided in the **Supplementary Methods** and **Figs. 2.1s–2.6s** , based on individual confusion matrices. Furthermore, values from these individual confusion matrices were used to calculate frame-based overall accuracy, precision, and recall for our system after initial model training and performance testing.

To assess segmentation accuracy, two blinded expert physicians retrospectively annotated 20–50 randomly selected clear frames per case as ground truth, focusing on key categories: normal pancreas, cysts, and solid tumors ( **Figs. 3.1s–3.3s** , **Table 2s** ). AI-generated segmentation masks for these frames were thus compared with expert annotations to evaluate model performance, assessed by calculating the Dice coefficient, which measures overlap accuracy, and Hausdorff distance, which evaluates boundary precision.

Clinical validation was performed on a third subset of patients included prospectively and consecutively (308 patients in total) as described below. Detection accuracy was assessed through real-time evaluations by conventional EUS and AI-EUS, requiring lesion presence or absence to be consistently indicated across ≥90% of frames in both spatial and temporal domains.

### EUS procedures

Procedures were performed using an Arietta processor (Fujifilm Healthcare Corp., Tokyo, Japan) with EG34-J10U and EG38-J10UT echoendoscopes (Pentax Medical, Montvale, New Jersey, USA). EUS was performed by two endoscopists (J.R., S.V.) with a lifetime experience of 20 000 and 30 000 procedures, respectively. Examinations were performed under propofol sedation administered by certified nurse anesthetists.

The study was approved by Orlando Health Institutional Review Board (IRB Approval Number-2066898). PANCRAIEUS software is currently not approved by the FDA and study registration was not mandated. All patients provided written informed consent for undergoing the EUS procedures. Given that only standard-of-care treatment was practiced based on EUS findings, with AI image analysis having no implications on patient care, the ethics committee waived requirement for separate study consent and exempted this study ( **Supplementary Methods** ).

EUS procedures were performed for each patient by one expert endoscopist (procedural endoscopist), who performed an examination of the pancreaticobiliary system adopting a station-based approach 20 . EUS images were simultaneously displayed on two separate monitors where one monitor displayed conventional EUS images and a second monitor displayed AI system indicators, such that each patient underwent assessment by both conventional EUS and AI-EUS, thereby acting as their own control. The procedural endoscopist was blinded to marks made by the AI system. The AI-EUS monitor was placed 3 m away from the procedural endoscopist, out of view, and was visualized by a second observing endoscopist who then documented the findings of AI system ( **Fig. 4s** ). This procedure room setting ensured that both endoscopists remained blinded to the alternative assessment. There was no acoustic signal emanating from the AI system.

Intraprocedural interventions and clinical care were performed based only on the EUS findings of the procedural endoscopist. To minimize bias, no pictures were captured until completion of the diagnostic station-based examination and the observing endoscopist had departed the procedure room to document the AI-EUS findings. The procedural endoscopist then sampled all solid mass lesions irrespective of size and cystic lesions ≥30 mm as per American Gastroenterological Association guidelines 21 . All procured specimens were interpreted by rapid on-site evaluation (ROSE) using previously described methods, and the final diagnosis for solid masses was established on histological assessment of cell blocks 22 . The final diagnosis for cyst aspirates was based on cytological findings at ROSE and in conjunction with chemistry and molecular profiling. The procedural endoscopist documented findings at EUS, ROSE, laboratory and histological assessments. All procedural evaluations including AI and conventional EUS were video recorded.

For AI-EUS, correct detection of the pancreatic lesion (true positive) was defined as presence of a solid mass or cystic lesion that was consistent in both spatial and temporal domains, over ≥90% of frames. A true-negative result was defined as the absence of detection of a solid mass or cystic lesion across both spatial and temporal domains over multiple frames, in correlation with the absence of a lesion on conventional EUS examination. Alternatively, a false-positive result was defined as detection of solid mass or cystic lesion on AI-EUS within an area observed to be normal pancreatic parenchyma at conventional EUS, and a false-negative result was defined as absence of detection of a solid mass or cystic lesion by AI-EUS despite presence of lesion at conventional EUS. Documented findings of the procedural endoscopist and observing endoscopist were correlated by a statistician.

The definition of ground truth for solid and cystic lesions is explained in the **Supplementary Material** . The study focus was detection (including segmentation) and not classification or final diagnosis of pancreatic solid masses or cystic lesions at EUS.

### Outcome measures

Although not a randomized trial, outcome measures were defined to provide clarity and structure to the observations. The primary outcome measure was detection and segmentation of solid mass lesions by AI-EUS. Secondary outcome measures were detection and segmentation of cystic lesions and normal pancreas by AI-EUS.

### Statistical analysis

This was a prospective, nonrandomized study to compare the rate of detection of pancreatic lesions between standard EUS and AI-EUS. Important details on sample size calculation 23 , summary, and analysis of patient and lesion characteristics, operating characteristics, and multivariable logistic regression analysis are provided in the **Supplementary Material** .

Two-sided *P* values were reported for comparison of all outcome measures and no adjustments were made for multiple testing. Statistical significance was determined as *P* < 0.05. All statistical analyses were performed using Stata 17.0 (Stata Corp., College Station, Texas, USA).

## Results

Between January 29, 2024, and July 5, 2024, 330 patients were screened for eligibility; 22 patients were excluded, due to altered gastric anatomy in 8, incomplete procedural examination in 5, and duodenal or esophageal stricture in 9. Patient and lesion characteristics of the 308 patients included in the study are shown in **Table 3s** .

The EUS examination of the pancreas revealed solid masses in 105 patients (34.1%), cystic lesions in 104 (33.8%), and normal evaluations in 99 (32.1%) ( Table 2 ). The final diagnosis of solid mass lesions was adenocarcinoma in 75, neuroendocrine tumor in 11, metastatic lesion to the pancreas in 3, lymphoma in 2, solid pseudopapillary neoplasm in 1, gastrointestinal stromal tumor in 1, and benign lesions in 12 patients.

**Table:** **Table 2** Patient details and characteristics of solid and cystic pancreatic lesions.

	Solid pancreatic mass lesions (n=105)		Pancreatic cystic lesions (n=104)	
Age, years				
Mean (SD)	67.0 (11.6)		67.8 (13.4)	
Median (IQR)	73 (13)		71.5 (15)	
Sex n (%)				
Female	47 (44.8)		51 (49.0)	
Male	58 (55.2)		53 (51.0)	
Lesion size, mm				
Mean (SD)	31.1 (14.9)		34.7 (26.2)	
Median (IQR)	30 (20)		25 (25)	
Lesion size, n (%)				
≤15 mm	11 (10.5)		27 (26.0)	
16–29 mm	26 (24.8)		34 (32.7)	
≥30 mm	68 (64.8)		43 (41.3)	
Total number of lesions				
Mean (SD)	1.1 (0.41)		1.6 (1.2)	
Median (IQR)	1 (0)		1 (1)	
Location of largest lesion, n (%)				
Head/uncinate process	58 (55.2)		31 (29.8)	
Genu/body/tail	47 (44.8)		73 (70.2)	
Final diagnosis, n (%)				
	Adenocarcinoma	75 (71.4)	Cystic NET	1 (1.0)
	GIST	1 (1.0)	Branch duct-IPMN	69 (66.4)
	Lymphoma	2 (1.9)	Mixed type-IPMN	3 (2.9)
	Metastatic lesion to the pancreas	3 (2.9)	Main duct IPMN	1 (1.0)
	NET	11 (10.5)	Pseudocyst	27 (26.0)
	Solid pseudopapillary neoplasm	1 (1.0)	Serous cystadenoma	3 (2.9)
	Benign lesion ^1^	12 (11.4)		
GIST, gastrointestinal stromal tumor; IPMN, intraductal papillary mucinous neoplasm; IQR, interquartile range; NET, neuroendocrine tumor. ^1^ Benign lesion types: accessory spleen (n = 1), autoimmune pancreatitis (n = 1), benign pancreatic parenchyma (n = 2), chronic pancreatitis (n = 8).				

### Pancreatic mass lesions

Demonstration of solid mass lesion by artificial intelligence-enhanced endoscopic ultrasonography, with confirmation of diagnosis of adenocarcinoma by rapid on-site assessment and histology.Video 1

The indication for EUS was presence of pancreatic mass on imaging in 70 (66.7%), presence of pancreatic cyst on imaging in 10 (9.5%), and clinical suspicion of pancreatic malignancy without definitive evidence of a lesion on cross-sectional imaging in 25 patients (23.8%).

The detection rate of solid mass lesions by AI-EUS was 97.1% (95%CI 91.9 to 99.4) compared with 100% (95%CI 96.6 to 100) by conventional EUS, resulting in a risk difference of 2.9% (95%CI –1.2 to 6.8), with no significant difference between AI-EUS and conventional EUS ( *P* = 0.25) ( Table 3 , Video 1 ). The detection rates of solid mass lesions by AI-EUS according to mass size were 81.8% (95%CI 48.2 to 97.7) for ≤15 mm, 100% (95%CI 86.8 to 100) for 16–29 mm, and 98.5% (95%CI 92.1 to 100) for ≥30 mm, with no significant difference in detection rates compared with conventional EUS.

**Table:** **Table 3** Rate of detection of correct lesion type on conventional endoscopic ultrasonography (EUS) and artificial intelligence-enhanced EUS examination.

Lesion type	Conventional EUS ^1^ n (%) [95%CI]	AI-EUS n (%) [95%CI]	Difference (95%CI)	*P*
No lesion present	99 (100) [96.3 to 100]	99 (100) [96.3 to 100)	0 (–2.7 to 2.7)	0.99
Pancreatic mass lesions				
All	105 (100) [96.6 to 100]	102 (97.1) [91.9 to 99.4]	2.9 (–1.2 to 6.8)	0.25
Size ≤15 mm	11 (100) [71.5 to 100]	9 (81.8) [48.2 to 97.7] ^2^	18.2 (–11.7 to 42.5)	0.48
Size 16–29 mm	26 (100) [86.8 to 100]	26 (100) [86.8 to 100]	0 (–9.7 to 9.7)	0.99
Size ≥30 mm	68 (100) [94.7 to 100]	67 (98.5) [92.1 to 100] ^3^	1.5 (–3.4 to 6.2)	0.99
Pancreatic cystic lesions				
All	104 (100) [96.5 to 100]	96 (92.3) [85.4 to 96.6]	7.7 (1.9 to 13.2)	0.007
Size ≤15 mm	27 (100) [87.2 to 100]	21 (77.8) [57.7 to 91.4] ^4^	22.2 (3.8 to 37.6)	0.02
Size 16–29 mm	34 (100) [89.7 to 100]	33 (97.1) [84.7 to 99.9] ^5^	2.9 (–6.4 to 12.0)	0.99
Size ≥30 mm	43 (100) [91.8 to 100]	42 (97.7) [87.7 to 99.9] ^6^	2.3 (–5.2 to 9.6)	0.99
AI, artificial intelligence; BD-IPMN, branch-duct intraductal papillary mucinous neoplasm; EUS, endoscopic ultrasonography. ^1^ Conventional EUS is the gold standard for comparison with AI-EUS. Lesions not detected on AI-EUS: ^2^ Adenocarcinoma (n = 2; one 11-mm mass in the body, one 15-mm mass in the head). ^3^ Neuroendocrine tumor (n = 1, 30-mm mass in the tail). ^4^ BD-IPMN (11-mm cyst in the genu [n = 1], 13-mm cyst in the body [n = 1], 14-mm cyst in the tail [n = 1], 15-mm cysts [n = 3; one cyst in the genu, two cysts in the tail]). ^5^ Pseudocyst (n = 1, 28-mm cyst in the genu). ^6^ BD-IPMN (n = 1, 30-mm cyst in the uncinate process).				

No false positives of solid pancreatic masses were observed from nonpancreatic structures when a consistent finding across ≥90% of frames in both spatial and temporal domains was considered. Three false negatives were observed in the AI-EUS findings; two lesions were adenocarcinomas and one was a neuroendocrine tumor ( Table 3 ). There was no significant difference in operating characteristics between conventional EUS and AI-EUS ( Table 4 ). No solid mass lesions identified by AI-EUS were missed at conventional EUS examination.

**Table:** **Table 4** Operating characteristics of conventional endoscopic ultrasonography (EUS) and artificial intelligence-based EUS for detection of solid pancreatic mass lesions.

Pancreatic mass	Conventional EUS ^1^	AI-EUS	*P*
All (n = 105)			
True positive	105	102	–
False positive	0	0	–
False negative	0	3	–
True negative	99	99	–
Sensitivity, % (95%CI)	100 (96.55 to 100)	97.14 (91.88 to 99.41)	0.25
Specificity, % (95%CI)	100 (96.34 to 100)	100 (96.34 to 100)	0.99
PPV, % (95%CI)	100 (96.55 to 100)	100 (96.45 to 100)	0.99
NPV, % (95%CI)	100 (96.34 to 100)	97.06 (91.64 to 99.39)	0.25
Accuracy, % (95% CI)	100 (98.21 to 100)	98.53 (95.76 to 99.70)	0.25
Size ≤15 mm (n = 11)			
True positive	11	9	–
False positive	0	0	–
False negative	0	2	–
True negative	99	99	–
Sensitivity, % (95%CI)	100 (71.51 to 100)	81.82 (48.22 to 97.72)	0.48
Specificity, % (95%CI)	100 (96.34 to 100)	100 (96.34 to 100)	0.99
PPV, % (95%CI)	100 (71.51 to 100)	100 (66.37 to 100)	0.99
NPV, % (95%CI)	100 (96.34 to 100)	98.02 (93.03 to 99.76)	0.50
Accuracy, % (95%CI)	100 (96.70 to 100)	98.18 (93.59 to 99.78)	0.50
Size 16–29 mm (n = 26)			
True positive	26	26	–
False positive	0	0	–
False negative	0	0	–
True negative	99	99	–
Sensitivity, % (95%CI)	100 (86.77 to 100)	100 (86.77 to 100)	0.99
Specificity, % (95%CI)	100 (96.34 to 100)	100 (96.34 to 100)	0.99
PPV, % (95%CI)	100 (86.77 to 100)	100 (86.77 to 100)	0.99
NPV, % (95%CI)	100 (96.34 to 100)	100 (96.34 to 100)	0.99
Accuracy, % (95%CI)	100 (97.09 to 100)	100 (97.09 to 100)	0.99
Size ≥30 mm (n = 68)			
True positive	68	67	–
False positive	0	0	–
False negative	0	1	–
True negative	99	99	–
Sensitivity, % (95%CI)	100 (94.72 to 100)	98.53 (92.08 to 99.96)	0.99
Specificity, % (95%CI)	100 (96.34 to 100)	100 (96.34 to 100)	0.99
PPV, % (95%CI)	100 (94.72 to 100)	100 (94.64 to100)	0.99
NPV, % (95%CI)	100 (96.34 to 100)	99.0 (94.55 to 99.97)	0.99
Accuracy, % (95%CI)	100 (97.82 to 100)	99.40 (96.71 to 99.98)	0.99
AI, artificial intelligence; EUS, endoscopic ultrasonography; NPV, negative predictive value; PPV, positive predictive value. ^1^ Conventional EUS is the gold standard for comparison with AI-EUS.			

### Pancreatic cystic lesions

Demonstration of cyst by artificial intelligence-enhanced endoscopic ultrasonography, with confirmation of diagnosis of mucinous lesion by rapid on-site assessment.Video 2

The indication for EUS was presence of pancreatic cyst on imaging in 77 (74.0%) and clinical suspicion of pancreatic malignancy in 27 patients (26.0%). Of 104 pancreatic cystic lesions, conventional EUS detected significantly more cysts than AI-EUS (100% [95%CI 96.5 to 100] vs. 92.3% [95%CI 85.4 to 96.6], risk difference 7.7% [95%CI 1.9 to 13.2]). There was a positive correlation between cyst size and detection by AI-EUS ( Table 3 , Video 2 ), with significantly lower detection rates for cysts ≤15 mm (77.8% [95%CI 57.7 to 91.4] vs. 100% [95%CI 87.2 to 100]). No false positives for pancreatic cysts were observed from nonpancreatic structures when a consistent finding across ≥90% of frames in both spatial and temporal domains was considered. Eight false negatives were observed in the AI-EUS findings: branch-duct intraductal papillary mucinous neoplasms (six 11–15 mm, one 30 mm), and one 28-mm pseudocyst. No cystic lesions identified by AI-EUS were missed at standard EUS examination. Operating characteristics for pancreatic cysts are shown in **Table 4s** .

On multivariable logistic regression analysis to predict factors associated with failure of cyst detection by AI-EUS, cyst size ≤15 mm was associated with detection failure when adjusted for lesion number and location ( **Table 5s** ).

### Normal pancreas and segmentation metrics

The results for detection of normal pancreas and segmentation metrics are shown in the **Supplementary Methods** and **Figs. 3.2s–3.3s** .

## Discussion

In the present study, we demonstrated the ability of a novel AI system – PANCRAIEUS – for detection and segmentation of solid mass lesions in the pancreas in routine clinical practice, and found its performance to be not significantly different to that of expert endoscopists.

Detection of solid mass lesions was chosen as the primary outcome because of the potential for significant variability in sonographic interpretation among endoscopists and important clinical implications of a missed diagnosis. We were able to not only demonstrate high sensitivity for detection of solid masses in real time but also correlate findings histologically in all patients. The robustness of technology is evident from the fact that a quarter of these patients did not have definitive evidence of solid neoplasm on cross-sectional imaging.

In previous reports, deep learning architectures were used to develop AI systems for diagnosis of pancreatic diseases on EUS images 24 25 and for station recognition 17 . While diagnostic sensitivity of 90%–96% was reported for adenocarcinoma, which is comparable to that achieved by EUS-guided tissue acquisition, these retrospective data were derived from a highly skewed cohort of patients with confirmed pancreatic diseases 24 25 . In addition, when retrospective assessments are performed using CNN models, other overlays from EUS processors are removed beforehand, resulting in superior performance that may be difficult to replicate in real-time clinical practice. The present study is different from others in that the reported findings were derived from a prospective cohort of patients who were evaluated in real time in a routine clinical setting.

Although not the primary objective, the detection of pancreatic cystic lesions by AI-EUS was also evaluated. While diagnostic accuracy was 99.3% for cysts >15 mm, performance was suboptimal for identifying cysts ≤15 mm; this may be due to a possible training mismatch, as vessels have the same appearance in transverse section as small cysts, representing a limitation of the current AI system. However, this limitation can be easily circumvented by color Doppler/power Doppler, including e-flow to clearly differentiate between small cysts and vessels. In addition, we believe that with the incorporation of additional training data in the future (including systematic usage of color Doppler/power Doppler), diagnostic performance for detection of small pancreatic cystic lesions can be improved. Moreover, the Dice similarity coefficient reported in this study are superior, as values are usually between 0.8 and 0.9 for both CT and MRI of the pancreas.

Given these study observations, how can the use of AI potentially impact the application of EUS for evaluation of the pancreas? Owing to complex regional anatomy, the pancreas is the most challenging organ to identify and hence lesions are difficult to sample at EUS. Use of AI could facilitate identification of the organ and easier detection of lesions for tissue sampling, particularly small cancers. In the present study, novel AI-EUS software identified the pancreas in all patients and detected solid lesions as small as 5 mm. With more training data, this performance can likely be further improved to >99%. Given the long learning curve of EUS examination and the lack of standardized and sufficient training modules, diagnostic ability varies among endoscopists, particularly in beginners 13 14 . This limitation can be ameliorated by incorporating AI in EUS, as it is likely to decrease the learning curve and thereby improve the technical performance of novice endoscopists. In a recent randomized trial, the use of AI during EUS increased diagnostic accuracy for detection of solid pancreatic lesions in novice endoscopists from 69% to 90%, although no significant improvement was observed in experienced endoscopists 26 . In addition, clear delineation of solid pancreatic masses may increase confidence in decisions to perform intraprocedural EUS-guided fine-needle biopsy. Often it can be difficult to differentiate lobularity in chronic pancreatitis from solid pancreatic neoplasm. Better identification of normal pancreas, cysts, and solid tumors is based on depiction of subtle gray-scale differences (the human eye can only differentiate 16–32 gray-scale levels compared with 256 shades displayed on ultrasound). The application of AI could potentially help discern these conditions and thereby facilitate selective targeting of lesions without compromising clinical outcomes. Importantly, areas mimicking masses, such as lobulation in pancreatitis, can be discerned more reliably by AI and an unnecessary biopsy can be obviated. By training the model to recognize different anatomical stations, potential blind spots in the pancreas can be avoided, resulting in more thorough examinations, which is an important criterion for quality control. This is particularly relevant as adjacent organs such as the gallbladder or large renal cysts that overlay the pancreas can mimic pancreatic cysts. In addition, the AI-EUS system can potentially streamline workflow by automating the segmentation process, saving time for medical staff, and possibly reducing procedural duration. However, over-reliance on AI-EUS by novice endoscopists has the potential to attenuate EUS skills and can negatively impact clinical outcomes, as AI may not be 100% accurate in all examinations.

The present study has several limitations. First, we did not include any clinical data within the AI model, which potentially could further enhance its performance and improve clinical workflows. Second, as the study was undertaken at a single center with only expert endosonographers, its robustness will require external clinical validation. In addition, as all participants in the study were referred to undergo EUS examination, there is potential for selection bias, which may affect the generalizability of study results. A multicenter study is currently in development. Third, training data and clinical validation were undertaken using a single EUS processor. Therefore, performance of PANCRAIEUS with other EUS processors is unclear and its testing is currently under way at our unit using all EUS processor types and in the hands of endosonographers with varying degrees of experience. Fourth, while other retrospective studies have examined the role of EUS-CNN models to differentiate between diseases of the pancreas, such as autoimmune pancreatitis, chronic pancreatitis, and adenocarcinoma, the present study focused only on detection and segmentation of solid mass lesions and not characterization of lesions, which is particularly relevant for pancreatic cysts. We are presently collecting these data prospectively to enable lesion characterization, which will be the logical next step in advancement of AI-EUS. Future development of computer-aided differential diagnosis systems should include finer subclassification of challenging pancreatic conditions such as chronic pancreatitis and pancreatic steatosis. Fifth, the performance outside the pancreas structure remains a limitation requiring further multicenter validation, although a persistence function of the system was implemented to disregard erratic individual frames showing as false positives. Sixth, we acknowledge the potential for bias and overestimation due to retrospective frame selection for quantitative metrics. However, prospective real-time clinical assessment ensured unbiased detection results, supplemented by retrospective metrics providing quantitative validation.

In conclusion, our study demonstrated that performance of AI-EUS was not significantly different to that of experienced endosonographers for detection and segmentation of solid pancreatic mass lesions. By standardizing performance, AI-EUS may have the potential to optimize performance and standardize clinical outcomes in pancreatic cancer.

## Supplementary Material

Supplementary Material Zusatzmaterial
