# Supplementary material for: Prospective clinical validation of a novel artificial intelligence system for real-time detection of solid pancreatic masses during endoscopic ultrasonography
Source: Endoscopy. 2025 Oct 13;58(3):223–32. doi: 10.1055/a-2701-6530 (PMC12923297; doi:10.1055/a-2701-6530)
Supplement: Supplementary file 3 — Supplementary Material [file 10-1055-a-2701-6530_27178282.pdf]

SUPPLEMENTARY MATERIAL

Prospective clinical validation of a novel artificial intelligence system for real-time detection of solid pancreatic masses during endoscopic ultrasonography

Ji Young Bang, Adrian Săftoiu, Anca Udriştoiu, Lucian Gruionu, Elena Codruța Gheorghe, Gabriel Gruionu, Jayapal Ramesh, Charles Melbern Wilcox, Shyam Varadarajulu

Endoscopy | DOI 10.1055/a-2701-6530 | © The Author(s).

Contents

SUPPLEMENTARY METHODS....4

AI MODEL TRAINING....4

ANNOTATION....4

TRAINING....5

PERFORMANCE TESTING....5

CASE-BASED ANALYSIS....6

FRAME-BASED ANALYSIS....6

CLINICAL VALIDATION....7

DICE COEFFICIENT....7

HAUSDORFF DISTANCE....8

mAP@50....8

Definition of ground truth for solid and cyst lesions....8

Statistical analysis: Summary and analysis of study data and regression analysis....8

Comparison of conventional EUS and AI-EUS for identification of normal pancreas....9

SUPPLEMENTARY TABLES....10

Table 1s Details of frame-based analysis....10

Table 2s Computed Dice coefficient, Hausdorff distance, and mAP@50 average precision metrics....10

Table 3s Patient details and lesion types....10

Table 4s Operating characteristics of conventional EUS and AI-EUS for detection of pancreatic cyst lesions....11

Table 5s Multivariable logistic regression analysis to determine the factors associated with failure of cyst detection on AI-EUS....12

SUPPLEMENTARY FIGURES....13

Fig. 1.1s Flow chart of the process for the development and continuous training of the AI model using EUS video data....13

Fig. 1.2s The data annotation and training procedures....13

Fig. 1.3s Example of frames annotations....13

Fig. 2.1s Annotated batch of individual frames for an individual case with a cystic lesion (blue) surrounded by normal pancreatic tissue (green)....14

Fig. 2.2s AI predicted batch of the same individual frames for an individual case with a cystic lesion (blue) surrounded by normal pancreatic tissue (green)....14

Fig. 2.3s Confusion matrix displayed as a function of annotated batch versus AI predicted batch of individual frames for an individual case with a cystic lesion....15

Fig. 2.4s Annotated batch of individual frames for an individual case with a solid mass (red) surrounded by normal pancreatic tissue (green)....15

Fig. 2.5s AI predicted batch of the same individual frames for an individual case with a solid mass (red) surrounded by normal pancreatic tissue (green)....16

Endoscopy | DOI 10.1055/a-2701-6530 | © The Author(s).

Fig. 2.6s Confusion matrix displayed as a function of annotated batch versus AI predicted batch of individual frames for an individual case with a solid mass lesion. 16

Fig. 3.1s Flow chart of the process for clinical validation, including calculation of metrics for the segmentation process. 17

Fig. 3.2s Dice similarity coefficient. 17

Fig. 3.3s A Hausdorff distance of 0 indicates that the boundaries of the two masks perfectly coincide, when DICE coefficient = 1. When there is no overlap between the two masks, the Hausdorff is maximum and DICE coefficient = 0. Hausdorff distance is useful when boundary accuracy is important, such as in medical image segmentation where the exact location of boundaries matters (e.g. delineating tumor boundaries). 17

Fig. 4s Room setting during the EUS procedure with blinding of the two endoscopists. 18

Endoscopy | DOI 10.1055/a-2701-6530 | © The Author(s).

SUPPLEMENTARY METHODS

AI MODEL TRAINING

Model training used a computer-implemented AI algorithm based on EUS images (including multiparametric EUS imaging), used to detect, mark and classify pancreatic solid masses and cystic lesions, as well as normal pancreas (patent number WO/2024/180385). The algorithm utilizes machine learning techniques to analyze the EUS images and extract relevant information, such as the size, shape, and location of the pancreatic mass (tumor) or cystic lesion, as well as normal pancreas. This information is then used to make predictions about the necessity to perform EUS-guided FNA/B according to current protocols and guidelines. The process of model training was based on a continuous process based on stored EUS videos (**Fig. 1.1s**).

ANNOTATION

The annotation procedure involved the following steps (**Fig. 1.2s**):

1. **Movie Trimming**: The EUS video representing a case (an EUS procedure on a patient) was trimmed to 5–15 minutes relevant to the pancreas procedure, with both gray-scale and color or power Doppler imaging sequences.

2. **Movie Cropping**: The trimmed video is split in frames and the non-clinical part (margins) of the frames was cropped.

3. **Image Labelling**: Two expert EUS investigators labelled the EUS grey-scale and color Doppler movies with respect to identification of pancreas (uncinate, head, neck, body and tail), cystic pancreatic lesions and solid pancreatic masses. The gold standard for diagnosis of cystic and/or solid pancreatic lesions was achieved by EUS-FNA or EUS-FNB with rapid on-site assessment followed by histology. Individual images (frames) were uploaded into VGG Image Annotator (https://www.robots.ox.ac.uk/~vgg/software/via/) and cross-labelled by two independent expert EUS investigators (**Fig. 1.3s**).

   The two expert EUS investigators marked three classes (pancreas, solid tumors, cystic lesions) on 20-50 images for each case (patient). The selection of 20-50 images ensured coverage of all relevant structures in the patient's video. Moreover, cases were included prospectively and consecutively and included the whole spectrum of pancreas appearances (including normal, steatopancreas and various degrees of chronic pancreatitis), solid masses (pancreatic adenocarcinoma, pancreatic neuroendocrine neoplasm, pancreas metastases from other primary tumors, solid pseudopapillary neoplasms, intrapancreatic accessory spleen) and cystic lesions (pseudocysts, serous cystadenomas, mucinous cyst neoplasms). Also, all the other structures in the EUS movie (black background or air artifacts, liver, spleen, kidneys, gallbladder, adrenals, lymph nodes, etc.) were annotated as “none” and further used for the model training as the 4th class. Using VIA, the outer edge of the class structures was marked, and the annotated image was saved. The selection of 20-50 representative images ensured coverage of all relevant structures in the patient's video.

Endoscopy | DOI 10.1055/a-2701-6530 | © The Author(s).

TRAINING

The training procedure involved the following steps:

1. **Initial Training**: The annotated images, which have been meticulously marked by the two expert EUS investigators, are utilized to train the PANCRAIEUS AI system. The training was performed using a slower but more complex CNN segmentation architecture CNN2 based on Mask R-CNN algorithm.

2. **Active Learning**: To augment the training dataset, each patient is subjected to PANCRAIEUS trained by CNN2, which automatically segments the images into the three classes: pancreas, cyst, and solid tumor. A sequence of 150-250 segmented images per patient was selected. Labeled and segmented data are then prepared in a suitable format to be seamlessly integrated into the VGG image annotator (VIA) software. This step allows for further analysis and review, as well as potential refinement, of the annotated images. Subsequently, the two EUS investigators reviewed each segmented image and make necessary adjustments to the segmentation if any inaccuracies are detected.

3. **Re-training the final model**: The verified and refined images are then reintroduced into the training dataset, enabling the AI model to continually improve its segmentation accuracy and performance. The training was performed using a faster but less complex CNN segmentation architecture CNN1 based on YOLO v8 algorithm, including 150 patients (29586 frames).

PERFORMANCE TESTING

Performance testing was performed on 52 different patients (3127 frames). Distribution of the final diagnosis for the included patients was the following: 21 patients with normal pancreas, 12 patients with cystic lesions greater than 10mm and 19 patients with solid masses. The final diagnosis was established by experts based on EUS-guided FNA or FNB (for cystic lesions and solid masses), as well as follow-up of at least 6 months. Performance testing comprised the following steps:

1. **Patient Selection**: Consecutive patients were selected after finalising the model training, ensuring a balanced distribution of cases, with each patient's diagnosis verified through EUS-guided FNA or FNB (cystic lesions and solid masses) and a minimum follow-up period of six months, ensuring reliable diagnostic confirmation.

2. **Algorithm Testing**: The AI system was tested using two CNN algorithms (YOLO v8.0 and R-CNN) to classify each patient's condition based on EUS videos.

3. **Classification by Non-Clinicians**: Two non-clinician operators reviewed the movies and classified each AI enhanced patient case into one of three categories: normal pancreas, cystic lesion, or solid mass.

4. **Case-based Accuracy**: The AI's case-based classifications were compared to the final diagnoses, and assigned to one of four categories: true positive, true negative, false positive, or false negative.

5. **Frame Annotation:** A subset of 50-100 frames per patient was annotated by two medical experts to allow a detailed, frame-level comparison of AI predictions.

6. **Algorithm Re-evaluation on Frames**: The two CNN algorithms were re-applied to each annotated frame to assess individual frame predictions.

7. **Confusion Matrix Calculation**: Based on frame-level results, a confusion matrix was created for each patient, categorizing frames into true positive, true negative, false positive, and false negative outcomes.

8. **Metric Calculation**: For each CNN algorithm, the overall accuracy, precision, and recall were automatically computed for cystic lesions and solid masses.

Endoscopy | DOI 10.1055/a-2701-6530 | © The Author(s).

CASE-BASED ANALYSIS

Case-based analysis evaluated AI's performance at a patient level, with both CNN algorithms (YOLO and Mask-RCNN) tested on EUS movies. The movies tested with two CNNs algorithms (YOLO and Mask-RCNN) were further categorized by two non-clinician operators (AU, LG) into the same diagnostic categories (normal pancreas, cystic lesions and solid masses) in order to derive case-based metrics of the overall accuracy for AI algorithms as compared to the final diagnosis. Based on acquired data, we compared the percentage (ratio) of AI-EUS procedures that yield clinically relevant information for the detection of cystic and/or solid pancreatic lesions in a typical patient population submitted for pancreas EUS in a case-based analysis as compared to the ground truth (final diagnosis obtained by EUS-guided FNA/FNB). For the case-based analysis, the following categories were considered:

- True positive: patients with cystic lesion or solid mass correctly identified as having it
- True negative: patients without cystic lesion or solid mass correctly identified as not having it
- False positive: patients without cystic lesion or solid mass incorrectly identified as having it
- False negative: patients with cystic lesion or solid mass incorrectly identified as not having it

The overall accuracy for case-based analysis was 100% (YOLO) & 91% (Mask-RCNN) for cystic pancreatic lesions, and 100% (YOLO) & 92% (Mask-RCNN) for solid pancreatic masses, respectively.

FRAME-BASED ANALYSIS

Frame-based analysis provided a more granular evaluation by annotating 50-100 frames per patient and re-running both CNN algorithms. Accuracy, precision, and recall were calculated based on frame-level classifications. This frame-based analysis highlighted the challenges of detecting lesions at the frame level, particularly with recall, underscoring the complexity of accurate frame segmentation in clinical settings.

Batches of individual frames were annotated by experts and the same frames were predicted by the AI system. These annotated and predicted frame batches were used to calculate confusion matrices for each individual case. Specifically, annotated frames showed clearly marked regions (pancreas, cysts, solid masses, background/none), while AI-predicted frames displayed regions detected by the system. Confusion matrices generated from comparisons between annotated and AI-predicted frames provided the basis for computing the frame-based overall accuracy, precision, and recall.

In order to test the predicted metrics for both CNNs, we manually annotated 50 to 100 individual frames for each of the individual cases (52 patients), based on a similar methodology to the model training. We then re-ran two CNN algorithms on the same individual frames and calculated automatically the confusion matrix for each case. Based on the confusion matrix for each individual case we defined the following categories:

- True positive: frames with cystic lesions or solid mass correctly identified as having it
- True negative: frames without cystic lesion or solid mass correctly identified as not having it
- False positive: frames without cystic lesion or solid mass incorrectly identified as having it
- False negative: frames with cystic lesion or solid mass incorrectly identified as not having it

The overall accuracy, precision and recall for frame-based analysis were automatically calculated and reported: 77%, 91%, 57% (YOLO) and 80%, 72%, 63% (Mask-RCNN) for cystic lesions, and 77%, 83%, 62% (YOLO) and 75%, 75%, 72% (Mask-RCNN) for solid masses, respectively (**Table 1s**).

The method for annotation of batches of individual frames by experts and how the same frames were predicted by the AI system are shown in **Figs. 2.1s**, **2.2s**, **2.4s**, and **2.5s**.

Furthermore, these annotated and predicted frame batches were used to calculate confusion matrices for each individual case, as shown in **Figs. 2.3s** and **2.6s**. Specifically, annotated frames show clearly marked regions (pancreas, cysts, solid masses, background/none), while AI-predicted frames display regions detected by the system.

Endoscopy | DOI 10.1055/a-2701-6530 | © The Author(s).

Confusion matrices generated from comparisons between annotated and AI-predicted frames provided the basis for computing the frame-based overall accuracy, precision, and recall reported above.

CLINICAL VALIDATION

Clinical validation was accomplished prospectively in an IRB approved clinical study conducted over 6 months. During the AI clinical testing conducted as part of the study, 10064 frames were randomly selected for metrics computation, including the Dice coefficient, Hausdorff distance and mAP@50 average precision. The flowchart in **Fig. 3.1s** represents the workflow for computing these metrics, with the following steps:

1. **Case selection**: Cases were randomly allocated from the IRB-approved study dataset, which was prospectively obtained after model training and performance testing. This step ensures a representative or unbiased sample for analysis.

2. **Frame Selection**: Once the cases were selected, another random selection of 20-50 frames per case were extracted from the medical videos associated with each case. This subset was used for further processing, including annotation and segmentation.

3. **Frame Annotation**: Two experts not involved in the study, annotated the selected frames, marking specific regions of interest, such as the pancreas, solid masses and cysts. These annotations served as the "ground truth" for later comparisons.

4. **Frame Segmentation:** The AI-based segmentation algorithm, PANCRAIEUS, was used to predict the segmentation mask for the same frames.

5. **Metrics Calculation:** The Dice coefficient, Hausdorff distance and mAP@50 were computed by comparing the AI-generated segmentation masks with the ground truth annotations from the expert medical doctors.

DICE COEFFICIENT

To compute the Dice coefficient (also known as the Dice similarity coefficient or DSC) for an AI segmentation algorithm, the predicted segmentation mask was compared with the ground truth mask selected by experts. The Dice coefficient measures the overlap between two samples and is particularly useful in segmentation tasks to quantify the accuracy of predicted masks. Both masks should be binary (with values of either 0 or 1), representing the segmented regions (**Fig. 3.2s**)

The Dice coefficient is computed using the following formula:

$$Dice = \frac{2 \times |A \cap B|}{|A| + |B|}$$

where A is the predicted mask and B is the ground truth mask, $|A \cap B|$ represents the number of pixels where both masks overlap, and $|A|$ and $|B|$are the total number of pixels in the predicted and ground truth masks, respectively. A total of 10064 frames, corresponding to 20-50 frames per case, were used for the Dice coefficient computation.

Endoscopy | DOI 10.1055/a-2701-6530 | © The Author(s).

HAUSDORFF DISTANCE

The Hausdorff distance measures the maximum distance between the boundary points of two sets. It quantifies how far the predicted segmentation is from the ground truth at the boundary level. The Hausdorff distance between two sets A and B is defined as:

$$H(A,B) = max\left\{ \sup_{a \in A} \inf_{b \in B} d(a,b), \sup_{b \in B} \inf_{a \in A} d(a,b) \right\}$$

where (a, b) is the distance between points a and b, and the Hausdorff distance computes the largest distance from a point in one set to the closest point in the other set.

mAP@50

The mAP@50 (mean Average Precision at 50% IoU) metric was computed to evaluate the average precision across all classes in the test dataset. Precision reflects how accurately the model predicts the correct bounding boxes, balancing the true positives (correct detections) against the false positives (incorrect detections). For each class, the model's predictions are evaluated to calculate precision at various recall levels. The model accumulates average precision across all classes to compute the mean Average Precision (mAP). **Table 2s** shows the computed Dice coefficient, Hausdorff distance and mAP@50 average precision metrics.

During clinical validation, 10,064 frames were randomly selected for quantitative metrics computation, including Dice coefficient, Hausdorff distance and mAP@50 average precision. When quantitative metrics for segmentation was examined, mean dice similarity coefficient (DSC) was 0.8824 for the pancreas, 0.9140 for cyst lesions and 0.9055 for mass lesions. Also, mAP@50 (mean Average Precision at 50% IoU) metric was 0.87 for pancreas, 0.84 for solid tumors and 0.646 for cystic lesions (**Figs. 3.2s**, **3.3s**).

Definition of ground truth for solid and cyst lesions

For solid mass lesions, histological findings at cell block were criteria for ground truth. For cyst lesions ≥30mm, cytological findings at ROSE in conjunction with chemistry and molecular profiling were criteria for ground truth. For cyst lesions <30mm that were not sampled and for normal pancreas, ground truth was based on annotations provided by the procedural endoscopist. Also, for cysts <30mm and for identification of normal pancreas, any discrepancy between reported assessments were resolved by a third endoscopist (JYB) by review of recorded videos and consensus establishment among all three endoscopists.

Statistical analysis: Summary and analysis of study data and regression analysis

Conventional EUS performed by expert endosonographers is the gold standard for examination of the pancreas and hence rate of detection of solid mass lesions by conventional EUS was determined at 99%. Based on European Society of Gastrointestinal Endoscopy (ESGE) guidelines, we judged that detection rate of solid mass lesions by AI-EUS of at least 90% would be clinically acceptable. Assuming detection rate of solid mass lesions of 99% by expert endosonographers, non-inferiority margin was established at 9%. Therefore, to declare noninferiority of AI-EUS to conventional EUS, the upper bound of the two-sided confidence interval for the difference in pancreatic mass detection rate needed to be less that 9%. We thus estimated that a sample size of 105 patients with solid mass lesions undergoing examination using both conventional EUS and AI-EUS would be required at 99% power and a one-sided error rate of 0.025 (PASS 15 Power Analysis and Sample Size Software, NCSS, LLC., Kaysville, Utah, USA). As approximately 34% of patients presenting to our institution with suspected pancreatic lesions have solid pancreatic mass lesions confirmed on EUS, the sample size was estimated at 308 patients to ensure that adequate number of patients with solid mass lesions would be enrolled in the study.

Patient characteristics and features of pancreatic lesions were summarized as means (with standard deviation) or medians (with interquartile range) for continuous data (depending on distribution of data) and as frequencies and proportions for categorical data. Operating characteristics of AI-EUS were also calculated, with findings at

Endoscopy | DOI 10.1055/a-2701-6530 | © The Author(s).

conventional EUS taken as the gold standard diagnostic examination. For comparison of categorical data, Chi-square or Fisher's exact test were used as indicated, and two-sample t-test or the Wilcoxon rank-sum test were used as indicated for comparison of continuous data. Difference in proportions and 95% confidence intervals (CI) were calculated using the Agresti-Caffo method.

A backward selection stepwise multivariable logistic regression analysis (using alpha value of 0.05) was performed to identify factors associated with inability of AI-EUS to detect pancreatic cyst lesions. Clinically relevant variables such as pancreatic cyst lesion size, number and location of cyst lesions were included as predictor variables.

Comparison of conventional EUS and AI-EUS for identification of normal pancreas

The indication for EUS was presence of suspected pancreatic cyst or mass on imaging in 34 (34.3%), abdominal pain in 45 (45.5%), and pancreatic cancer screening in 20 patients (20.2%). There was no false positive detection of solid mass or cystic lesions by AI-EUS in patients with normal pancreas and hence there was 100% concordance between conventional EUS and AI-EUS for detection and segmentation of normal pancreas.

Endoscopy | DOI 10.1055/a-2701-6530 | © The Author(s).

SUPPLEMENTARY TABLES

Table 1s Details of frame-based analysis

Diagnosis	Overall accuracy (case-based)	Overall accuracy (frame based)	Precision	Recall
Cystic lesions CNN1	1	0.77	0.91	0.57
Solid masses CNN1	1	0.77	0.83	0.62
Cystic lesions CNN2	0.91	0.8	0.72	0.63
Solid masses CNN2	0.92	0.75	0.75	0.72

Table 2s Computed Dice coefficient, Hausdorff distance, and mAP@50 average precision metrics

**Tissue Type**	**Number of Frames**	**Median Dice Coefficient**	**Median Hausdorff Distance (pixels)**	**mAP@50**
Pancreas	6856	0.8824	68	0.87
Tumors	1762	0.9055	64	0.84
Cysts	1446	0.9140	34	0.646

Table 3s Patient details and lesion types

**Age (years):**	**Mean (SD)**	66.2 (14.3)
	**Median (IQR)**	69.5 (16)
**Gender: n (%)**	**Female**	163 (52.9)
	**Male**	145 (47.1)
**Race: n (%)**	**Asian**	3 (0.97)
	**Black**	33 (10.7)
	**Hispanic**	38 (12.3)
	**White**	234 (76.0)
**Lesion type: n (%)**	**No lesions present**	99 (32.1)
	**Solid mass lesions**	105 (34.1)
	**Pancreatic cyst lesions**	104 (33.8)

Endoscopy | DOI 10.1055/a-2701-6530 | © The Author(s).

Table 4s Operating characteristics of conventional EUS and AI-EUS for detection of pancreatic cyst lesions

		Conventional EUS	AI-EUS (PANCRAIEUS)	p-value
**Pancreatic cyst lesions: All** (n=104)	**True positive**	104	96	-
	**False positive**	0	0	-
	**False negative**	0	8	-
	**True negative**	99	99	-
	**Sensitivity, % (95% CI)**	100 (96.52 - 100)	92.31 (85.40 - 96.62)	0.007
	**Specificity, % (95% CI)**	100 (96.34 - 100)	100 (96.34 - 100)	0.999
	**PPV, % (95% CI)**	100 (96.52 - 100)	100 (96.23 - 100)	0.999
	**NPV, % (95% CI)**	100 (96.34 - 100)	92.52 (85.80 - 96.72)	0.007
	**Accuracy, % (95% CI)**	100 (98.20 - 100)	96.06 (92.38 - 98.28)	0.007
**Pancreatic cysts: Size ≤15 mm** (n=27)	**True positive**	27	21	-
	**False positive**	0	0	-
	**False negative**	0	6	-
	**True negative**	99	99	-
	**Sensitivity, % (95% CI)**	100 (87.23 - 100)	77.78 (57.74 - 91.38)	0.023
	**Specificity, % (95% CI)**	100 (96.34 - 100)	100 (96.34 - 100)	0.999
	**PPV, % (95% CI)**	100 (87.23 - 100)	100 (83.89 - 100)	0.999
	**NPV, % (95% CI)**	100 (96.34 - 100)	94.29 (87.98 - 97.87)	0.029
	**Accuracy, % (95% CI)**	100 (97.11 - 100)	95.24 (89.92 - 98.23)	0.029
**Pancreatic cysts: Size 16–29 mm** (n=34)	**True positive**	34	33	-
	**False positive**	0	0	-
	**False negative**	0	1	-
	**True negative**	99	99	-
	**Sensitivity, % (95% CI)**	100 (89.72 - 100)	97.06 (84.67 - 99.93)	0.999
	**Specificity, % (95% CI)**	100 (96.34 - 100)	100 (96.34 - 100)	0.999
	**PPV, % (95% CI)**	100 (89.72 - 100)	100 (89.42 - 100)	0.999
	**NPV, % (95% CI)**	100 (96.34 - 100)	99.0 (94.55 - 99.97)	0.999
	**Accuracy, % (95% CI)**	100 (97.26 - 100)	99.25 (95.88 - 99.98)	0.999
**Pancreatic cysts: Size ≥30 mm** (n=43)	**True positive**	43	42	-
	**False positive**	0	0	-
	**False negative**	0	1	-
	**True negative**	99	99	-
	**Sensitivity, % (95% CI)**	100 (91.78 - 100)	97.67 (87.71 - 99.94)	0.999
	**Specificity, % (95% CI)**	100 (96.34 - 100)	100 (96.34 - 100)	0.999
	**PPV, % (95% CI)**	100 (91.78 - 100)	100 (91.59 - 100)	0.999
	**NPV, % (95% CI)**	100 (96.34 - 100)	99.0 (94.55 - 99.97)	0.999
	**Accuracy, % (95% CI)**	100 (97.44 - 100)	99.30 (96.14 - 99.98)	0.999

Abbreviations: NPV, negative predictive value; PPV, positive predictive value.

Endoscopy | DOI 10.1055/a-2701-6530 | © The Author(s).

Table 5s Multivariable logistic regression analysis to determine the factors associated with failure of cyst detection on AI-EUS

**Multivariable logistic regression analysis**			
**Predictor variable**	**Odds ratio**	**95% CI**	**p-value**
**Size of cyst: ≤15 mm vs. >15 mm**	10.11	1.86 - 54.89	0.007
**Number of cysts: ≥2 vs. 1**	0.35	0.038 - 3.24	0.354
**Location of largest cyst: Genu/body/tail vs. Head/uncinate process**	3.28	0.35 - 30.33	0.295
**Backward selection stepwise multivariable logistic regression analysis**			
**Predictor variable**	**Odds ratio**	**95% CI**	**p-value**
**Size of cyst: ≤15 mm vs. >15 mm**	10.71	2.01 - 57.02	0.005

Endoscopy | DOI 10.1055/a-2701-6530 | © The Author(s).

SUPPLEMENTARY FIGURES

Fig. 1.1s Flow chart of the process for the development and continuous training of the AI model using EUS video data.

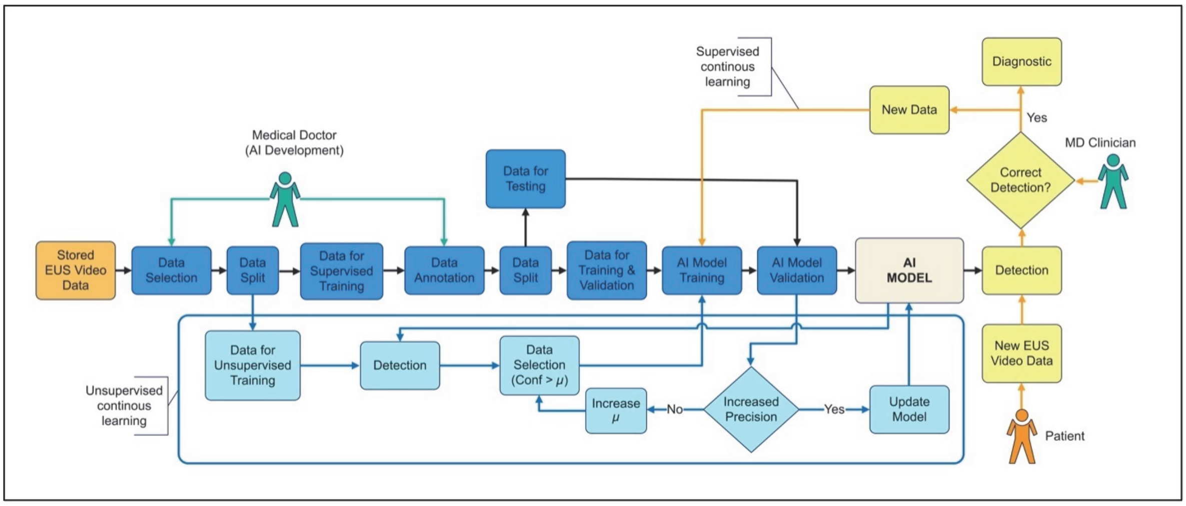

Fig. 1.2s The data annotation and training procedures.

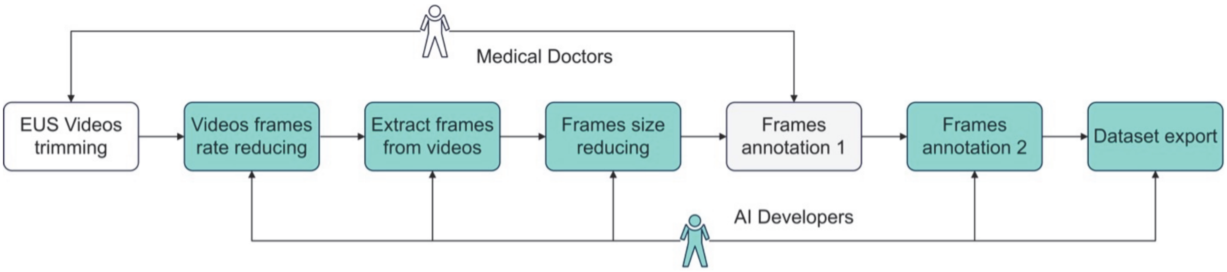

Fig. 1.3s Example of frames annotations.

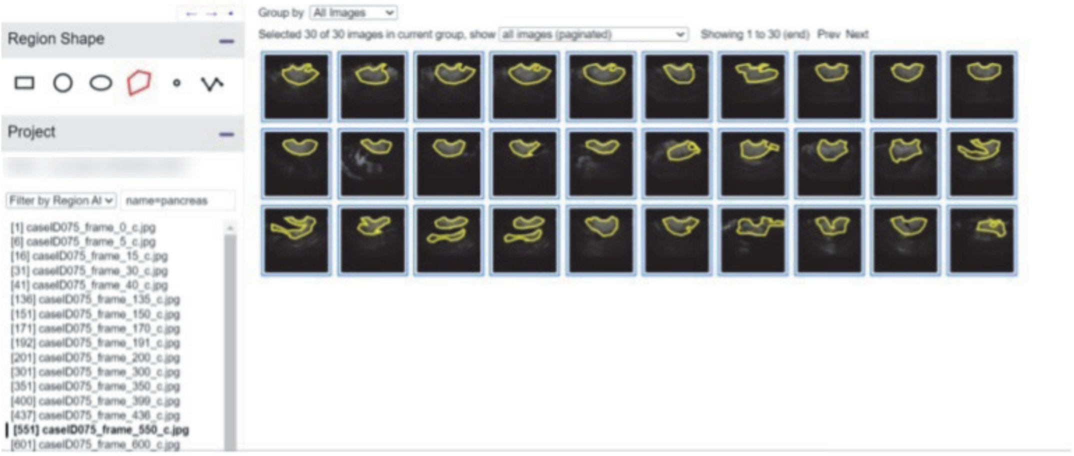

Endoscopy | DOI 10.1055/a-2701-6530 | © The Author(s).

Fig. 2.1s Annotated batch of individual frames for an individual case with a cystic lesion (blue) surrounded by normal pancreatic tissue (green).

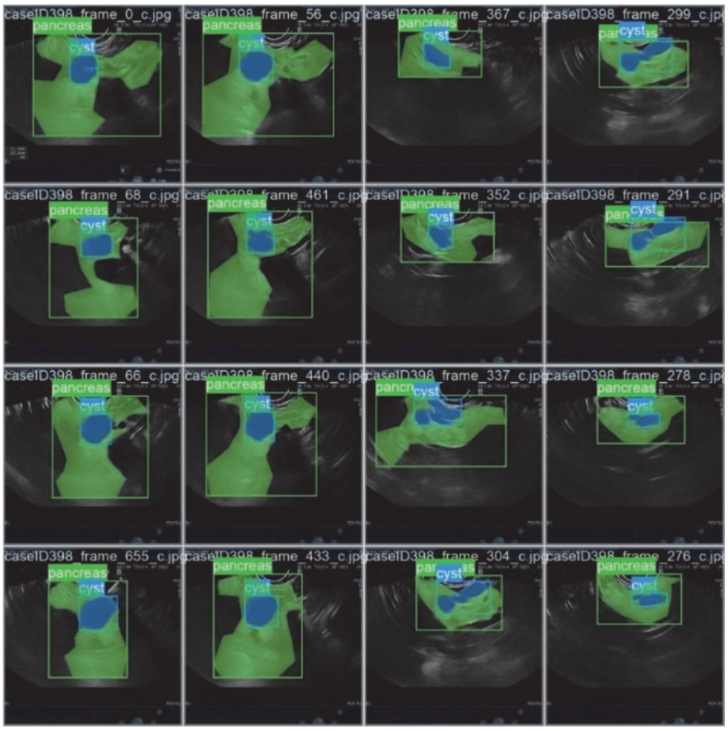

Fig. 2.2s AI predicted batch of the same individual frames for an individual case with a cystic lesion (blue) surrounded by normal pancreatic tissue (green).

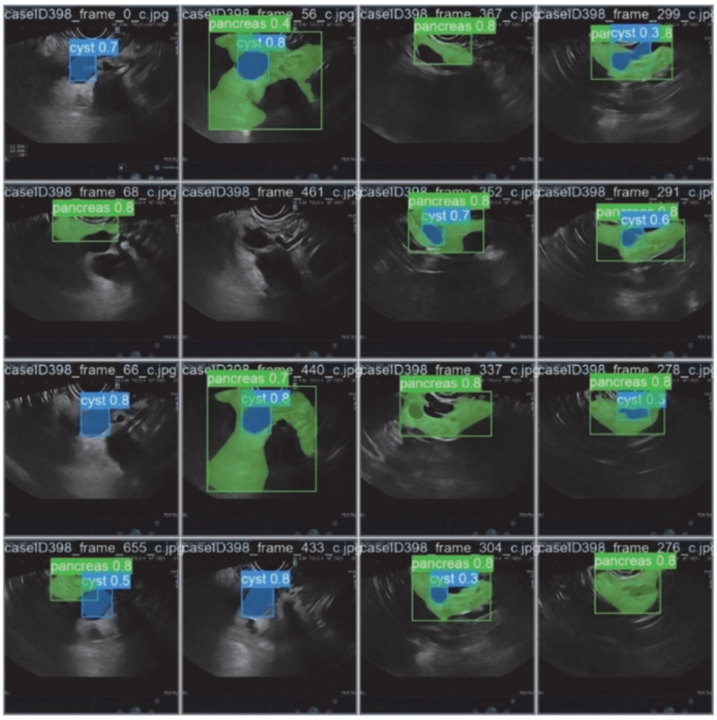

Endoscopy | DOI 10.1055/a-2701-6530 | © The Author(s).

Fig. 2.3s Confusion matrix displayed as a function of annotated batch versus AI predicted batch of individual frames for an individual case with a cystic lesion.

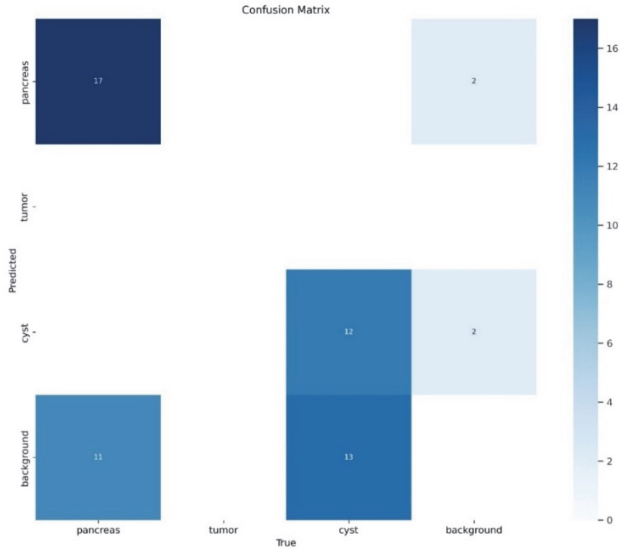

Fig. 2.4s Annotated batch of individual frames for an individual case with a solid mass (red) surrounded by normal pancreatic tissue (green).

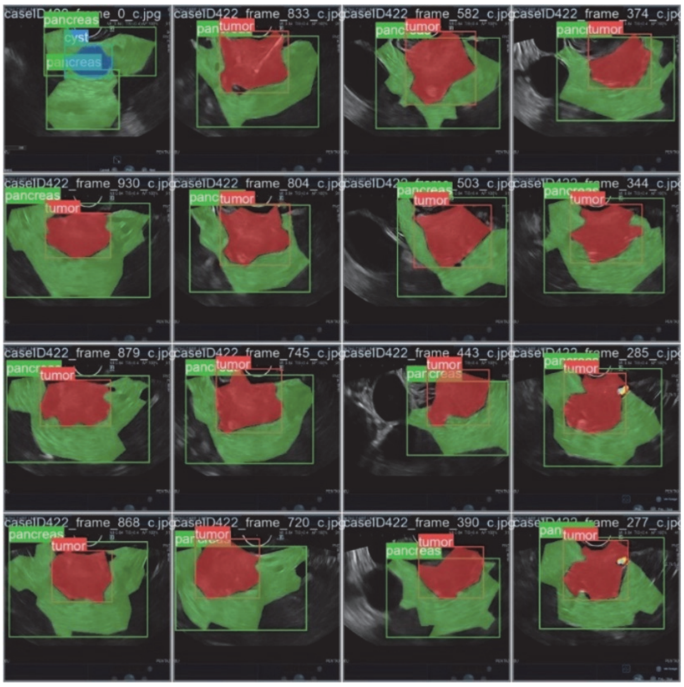

Endoscopy | DOI 10.1055/a-2701-6530 | © The Author(s).

Fig. 2.5s AI predicted batch of the same individual frames for an individual case with a solid mass (red) surrounded by normal pancreatic tissue (green).

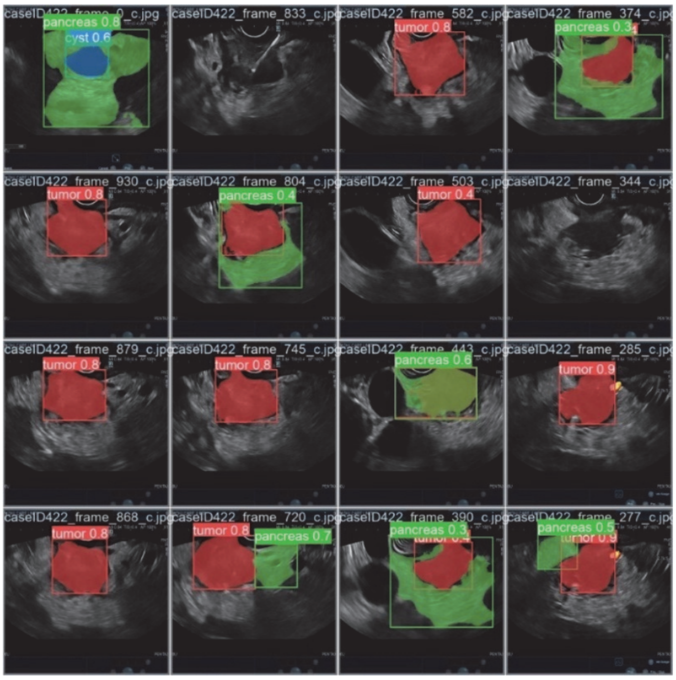

Fig. 2.6s Confusion matrix displayed as a function of annotated batch versus AI predicted batch of individual frames for an individual case with a solid mass lesion.

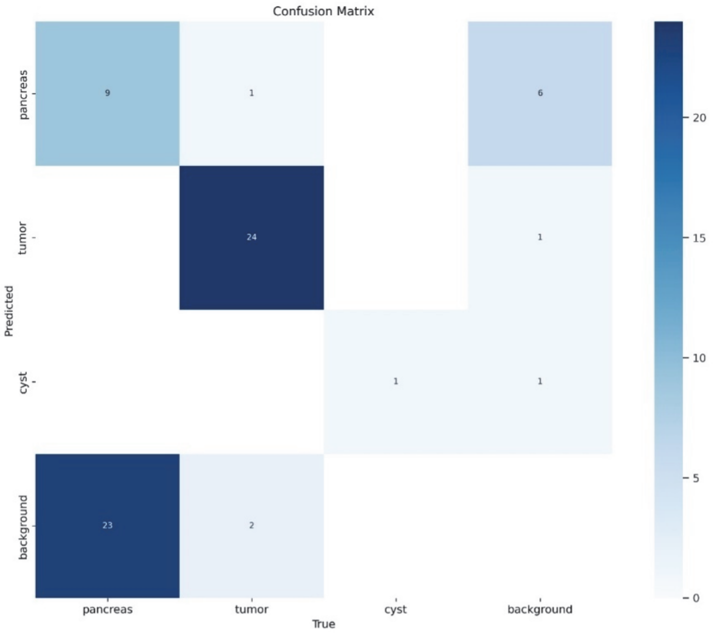

Endoscopy | DOI 10.1055/a-2701-6530 | © The Author(s).

Fig. 3.1s Flow chart of the process for clinical validation, including calculation of metrics for the segmentation process.

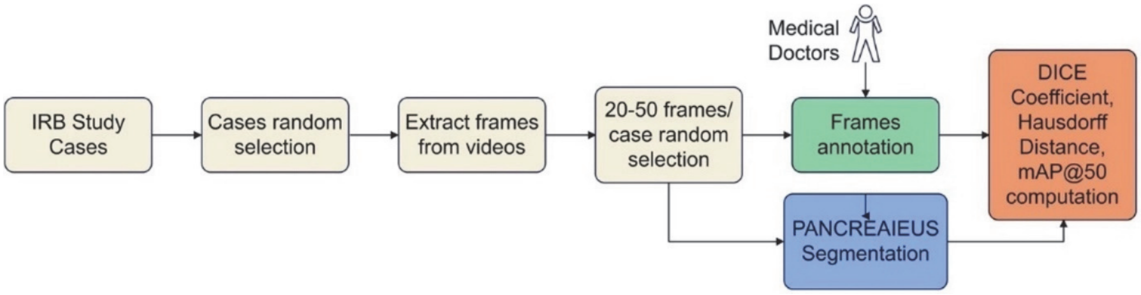

Fig. 3.2s Dice similarity coefficient.

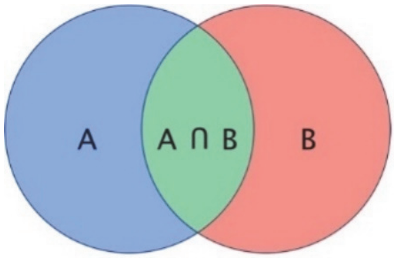

Fig. 3.3s A Hausdorff distance of 0 indicates that the boundaries of the two masks perfectly coincide, when DICE coefficient = 1. When there is no overlap between the two masks, the Hausdorff is maximum and DICE coefficient = 0. Hausdorff distance is useful when boundary accuracy is important, such as in medical image segmentation where the exact location of boundaries matters (e.g. delineating tumor boundaries).

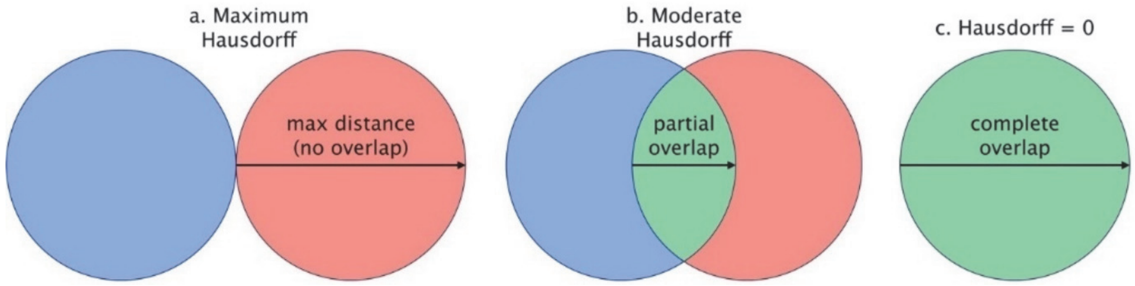

Endoscopy | DOI 10.1055/a-2701-6530 | © The Author(s).

Fig. 4s Room setting during the EUS procedure with blinding of the two endoscopists.

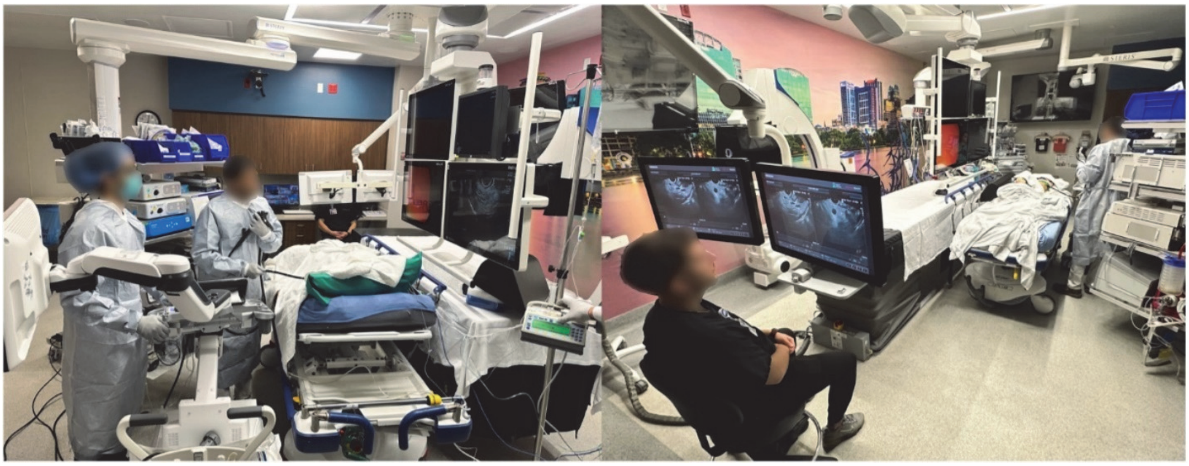

Endoscopy | DOI 10.1055/a-2701-6530 | © The Author(s).
